# Mathematical Model for Describing Corn Grain Dehydration Kinetics after a Nixtamalization Process

**DOI:** 10.3390/foods10081771

**Published:** 2021-07-30

**Authors:** Miguel Ángel Gruintal-Santos, María Teresa Zagaceta-Álvarez, Karen Alicia Aguilar Cruz, Juan Reséndiz-Muñoz, Héctor Eduardo Martinez-Flores, Jose Luis Fernández-Muñoz

**Affiliations:** 1 Universidad Autónoma de Guerrero, Facultad de Ciencias Agropecuarias y Ambientales, Unidad Tuxpan, km 2.5 Carretera Iguala-Tuxpan, Iguala de la Independencia 40101, Mexico; magruintal@uagro.mx; 2 Instituto Politécnico Nacional, Escuela Superior de Ingeniería Mecánica y Eléctrica, Unidad Azcapotzalco, Ciudad de México C.P. 02250, Mexico; mzagaceta@ipn.mx; 3 Instituto Politécnico Nacional, Centro de Investigación en Computación, Unidad Zacatenco, Ciudad de México C.P. 07738, Mexico; kaguilarc1400@alumno.ipn.mx; 4 Q&P Consulting, Unidad Azcapotzalco, Ciudad de México C.P. 02230, Mexico; resendizm1973@gmail.com; 5 Facultad de Químico-Farmacobiología, Universidad Michoacana de San Nicolás de Hidalgo, Morelia C.P. 58240, Mexico; hector.martinez.flores@umich.mx; 6 Instituto Politécnico Nacional, Centro de Investigación en Ciencia Aplicada y Tecnología Avanzada, Unidad Legaria, Ciudad de México C.P. 11500, Mexico

**Keywords:** asymptotic model, steeping time influence, grain hydration process, nixtamalization, isothermal dehydration

## Abstract

In this research, the mathematical model associated with the hydrothermal dehydration process of Nixtamalized Corn Grains (NCG) with different Steeping Time (ST) values, allows the fitting of experimental data with initial moisture $M_{0}$ and the equilibrium moisture $M_{E}$ as a function of Isothermal Dehydration Time (IDT). The moisture percentage for any time t and dehydration rate (isolines M(t) and isolines $v_{I}$ respectively) of the NCG is shown by means of matrix graphics as a simultaneous function of IDT and ST. The relationship between initial dehydration rate $v_{0}$ and initial moisture $M_{0}$ establishes as a function of ST. Also, the mathematical model associated with the solution of the second Fick’s law allows calculating the diffusivity rate $v_{k}$ (H_2_O molecules out of NCG) and verify that the rate of change in moisture and the dynamical proportionality constant k has a non-linear dependence on the IDT and that k is directly proportional to $D_{eff}$. The k values strongly relate to ST and the calcium ions percentage into NCG according to solubility lime values into cooking water (or nejayote) as a function of decreasing temperature when ST increases.

## 1. Introduction

Corn production worldwide is larger than any other cereal. The United States Department of Agriculture (USDA) estimates that the 2020/2021 World Corn Production would be 1133.89 million tons. Corn Production last year was 1116.41 million tons. The 1133.89 million tons estimated for this year could mean an increase of 17.47 million tons or 1.57% in the production of corn around the world. Among the main corn producers are: United States: 360,252,000 tons, China: 260,670,000 tons, Brazil: 109,000,000 tons, European Union: 63,600,000 tons Argentina: 47,500,000 tons, Ukraine: 29,500,000 tons, India: 28,500,000 tons, Mexico: 28,000,000 tons. 

The TNP, originally from Mexico, is an alkaline hydrothermal process that consisting of the cooked corn grains immersed in an aqueous solution of CaOH_2_ (“nejayote” in the indigenous language), which have three purposes: the pericarp degradation, the calcium ions diffusion, and hydration on corn grains. The alkaline aqueous solution is prepared from 0.8% up to 2% of lime with respect to the weight of corn grains, where temperatures can vary from 72 °C until the solution boils. Afterward, the corn grains submerge into the nejayote, which cools to room temperature according to Newton’s law of cooling. This process is named Steeping Time (ST) and must last several hours. Simultaneously, in the cooking time and ST stages, partial NCG-starch hydration, Ca^++^ and Ca(OH)^+^ ions (divalent and monovalent respectively) [1] diffusion increases, allowing the pre-gelatinization and starches gelatinization of the outermost endosperm part. In this way, the hydration process occurs faster. Later, the nejayote drained, and the NCG is three times water-rinsed to eliminate the residual lime, which, in turn, facilitates the NCG milling and the conversion to fresh corn dough [2,3]. Then, the NCG is milled in a stone mill to prepare fresh corn masa, and circular disks are molded to prepare tortillas. The NCG’s dehydration kinetics, after the TNP described above, reflects the physical-chemical state of water and the Ca^++^ and Ca(OH)^+^ ions into the grains, and the water extraction by heating must be applied with a temperature less than that of starch gelatinization during the isothermal dehydration process. The corn grains hydration process has been studied as a function of ST, such as the ST effect on different physical-chemical properties of the Instantaneous Nixtamalized Corn Flour and Ca^++^ and Ca(OH)^+^ ions diffusion through the different sections of the NCG: pericarp, endosperm, and germ [3,4,5]. Also, the absorption mechanism and the percolation channels for Ca^++^ and Ca(OH)^+^ ions diffusion to explain the accumulation process in the external layers of NCG have been studied. These ions contribute to the total calcium percentage in the corn grains [3,5,6,7].

In the past, for the alkaline hydration process, some mathematical models based on three parameters published to date: k is dynamical proportionality constant, $M_{S}$ maximum moisture percentage, saturation or equilibrium and $M_{0}$ endogenous moisture. For example, the differential Equation (1), in varieties of beans and fava beans, results in the fact that the water absorption ratio is directly proportional to the difference of the percentage of maximum moisture and the percentage of moisture at any time with a constant of proportionality k [6,7,8,9,10,11,12]. However, they omitted to indicate which was the experimental data to determine the border conditions and for solving the differential equation. Another researcher group proposed the differential Equation (2) for the water absorption ratio in red beans, which is proportional to the difference between the maximum moisture percentage and the moisture percentage at any time t [12,13,14]. They did not propose a value of k. Other authors proposed an asymptotic exponential model using Equation (3) to explain the water absorption kinetics in chickpeas, millets, cereal flakes, and soybeans [12,13,14,15,16,17,18]. Moreover, they did not justify their results mathematically or with experimental data.

(1) $$\frac{dM\left(t\right)}{dt}=-k\left(M-M_{S}\right)$$

(2) $$\frac{dM\left(t\right)}{dt}=-\left(M-M_{S}\right)$$

(3) $$M\left(t\right)=M_{S}+\left(M_{S}-M_{0}\right)e^{-k_{h}t}$$

A mathematical model was proposed, with corn grains spherical geometry, employing the second Fick’s law, to explain the Ca^++^ and Ca(OH)^+^ ions diffusion across the pericarp membrane and areas without pericarp [18]. The H_2_O-Ca simultaneous diffusion into corn grains without a pericarp is explained by employing the second Fick’s equation solution for chemical reactions during the TNP, in cooked isothermal alkaline [19,20]. Also, there are researches about calculating the barrier effects on pericarp of NCG in the water diffusion during an alkaline thermal treatment, and k values compare for NCG with no pericarp [20,21]. As far as is known, no evidence exists on the fitting of results during the dehydration process after TNP with a satisfactory explanation, using a mathematical model and its relationship with Fick’s law for the water diffusion as a function of ST. Fick’s equation can be related, with the fitting the NCG dehydration curves to calculate $D_{eff}$ and k values for water diffusivity or water evaporation of the NCG. Therefore, this research aims at deducing and solving the first-order differential equation to describe the dehydration kinetics of NCG after TNP at different ST, applying border conditions and the experimental data fitting to verify the mathematical model validity. Besides, the mathematical model calculates the moisture percentage and dehydration rate isolines. The model calculates k as a function of $D_{eff}$ during IDT.

## 2. Experimental

By performing TNP, the white corn grains using (a commercial variety called Toluqueño harvest 2019) with 14.54% average of endogenous moisture, and with the same qualitative characteristics such as not broken, similar grain sizes, and color. The mixture is prepared into an Erlenmeyer flask with 10 g of corn grains adding water until reaching 30 mL; besides, the mixture contains 0.8% of Ca(OH)_2_ with respect to the weight of the white corn grains (0.36 g of lime). Then, the mixture cooks at 80 °C for 60 min on a thermostat water bath (ECOSHEL, USA). The flask is covering with aluminum paper to avoid water evaporation. This process repeats five times for every ST (0, 2, 4, 6, and 8 h). Thus, the total NTP was 25 times. Then, the NCG was dried and weighed simultaneously without washing (into an equipment balance with a halogen lamp model HR83 Mettler-Toledo; Switzerland). The chosen time for dehydration in the 0–120 min interval at 50 °C (temperature to avoid starches gelatinization during dehydration process) constantly taking weight measurements every 15 min during the first 2 h. To the $M_{E}$ parameter, samples dehydrate for 2600 min. There were five measurements for each ST experiment.

## 3. Mathematical Model

For the NCG the solution to Equation (4) and its relationship with Fick’s law allows calculating the effective diffusivity coefficient during the isothermal dehydration process as a function of ST. The NCG loses water continuously. This loss is expressed by employing a moisture differential dM(t) per a time differential dt, where dM(t) is proportional to M(t) it is contained in NCG at any t plus a constant C where the constant depends on the border conditions obtained from experimental data.

(4) $$\frac{dM\left(t\right)}{dt}=-k\left[M\left(t\right)+C\right]$$

where k is a dynamical constant. The border conditions Equation (4) expressed by Equation (5):

(5) $$M\left(t\right)=\{\begin{array}{c} M_{0}\;\Leftrightarrow t\to 0\\ M_{E}\Leftrightarrow t\to \infty \end{array}$$

where $M_{0}$ and, $M_{E}$ are the initial moisture and equilibrium moisture, respectively [21]. The Isothermal Dehydration Process develops from conditions of Equation (5). Integrating Equation (4):

(6) $$\int_{M_{0}}^{M\left(t\right)}\frac{dM\left(t\right)}{M\left(t\right)+C}=-k\int_{0}^{t}dt$$

From Equation (6) the resulting is:

(7) $$ln\;\frac{M\left(t\right)+C}{M_{0}+C}=-kt$$

Then, the moisture percentage for any time is:

(8) $$\Rightarrow M\left(t\right)=\left(M_{0}+C\right)e^{-kt}-C$$

By substituting border conditions in Equation (8), obtains Equations (9) and (10)

(9) $$Lim{\;}_{t\to 0}\lfloor M\left(t\right)\rfloor =Lim{\;}_{t\to 0}\left[\left(M_{0}+C\right)e^{-kt}-C\right]=M_{0}$$

(10) $$Lim{\;}_{t\to \infty }\lfloor M\left(t\right)\rfloor =Lim{\;}_{t\to \infty }\left[\left(M_{0}+C\right)e^{-kt}-C\right]=-C=M_{E}$$

By substituting Equations (9) and (10) into Equations (4) and (8), respectively, obtains Equations (11) and (12), Where $v_{I}$ is the Isothermal Dehydration Rate for all times t.

(11) $$v_{I}=\frac{dM\left(t\right)}{dt}=-k\left[M\left(t\right)-M_{E}\right]$$

(12) $$M\left(t\right)=\left(M_{0}-M_{E}\right)e^{-kt}+M_{E}$$

Developing the term that corresponds to the moisture ratio $M_{R}$ is expressed by Equation (13) for the hydration process [21,22,23], and can also be expressed by the right part, which is the solution to the second Fick’s law

(13) $$M_{R}=\frac{M\left(t\right)-M_{E}}{M_{0}-M_{E}}=e^{-kt}=1-\frac{6}{\pi^{2}}\overset{\infty }{\underset{n=1}{\sum }}{\left(-1\right)}^{n}\frac{1}{n^{2}}\exp\left(-\frac{D_{eff}n^{2}\pi^{2}t}{a^{2}}\right)$$

where n is summation terms, a is corn grain radius, and $D_{eff}$ is an effective diffusivity coefficient.

Then Isothermal Dehydration Rate $v_{I}$ can be calculated using two methods: (1) from the first-derivative of Equations (12) and (2) by employing Equation (11). Therefore:

(14) $$v_{I}=\frac{\begin{array}{c} dM\left(t\right) \end{array}}{dt}=-k\left(M_{0}-M_{E}\right)e^{-kt}=-k\left(M_{0}-M_{E}\right)\overset{\infty }{\underset{n=0}{\sum }}\frac{{\left(-kt\right)}^{n}}{n!}\;\;\;$$

Border conditions for Equation (14) are shown in Equation (15) following experimental data.

(15) $$v_{I}=\frac{dM\left(t\right)}{dt}=\{\begin{array}{c} v_{0}\Leftrightarrow t\to 0\\ 0\Leftrightarrow t\to \infty \end{array}$$

Therefore, Equations (16) and (17) are:

(16) $$Lim_{t\to 0}\left[\frac{dM\left(t\right)}{dt}\right]=Lim_{t\to 0}\left[-k\left(M_{0}-M_{E}\right)e^{-kt}\right]=-k\left(M_{0}-M_{E}\right)=v_{0}$$

(17) $$Lim_{t\to \infty }\left[\frac{dM\left(t\right)}{dt}\right]=Lim_{t\to \infty }\left[-k\left(M_{0}-M_{E}\right)e^{-kt}\right]=0$$

where $v_{0}$ is the initial dehydration rate, and which one will be applied to experimental results. In another analysis, t obtains from Equation (12) to give Equation (18).

(18) $$t=\frac{1}{k}Ln\left[\frac{1}{M_{R}}\right]=\frac{1}{k}Ln\left[\frac{M_{0}-M_{E}}{M\left(t\right)-M_{E}}\right]=\frac{a^{2}}{\;\;\pi^{2}D_{eff}}Ln\left[\frac{M_{0}-M_{E}}{M\left(t\right)-M_{E}}\right]$$

To explain the isothermal dehydration process adequately, to the Equations (19) and (20), there are limits.

(19) $$Lim{\;}_{t\to \infty }\lfloor M\left(t\right)\rfloor =Lim{\;}_{t\to \infty }\left[\left(M_{0}-M_{E}\right)e^{-kt}+M_{E}\right]=M_{E}$$

(20) $$Lim{\;}_{M\left(t\right)\to M_{E}}v_{I}=Lim{\;}_{M\left(t\right)\to M_{E}}\frac{dM\left(t\right)}{dt}=Lim{\;}_{M\left(t\right)\to M_{E}}\left(-k\left[M\left(t\right)-M_{E}\right]\right)=0\;$$

(21) $$Ln\left[\frac{dM\left(t\right)}{dt}\right]=Ln\left[-k\left(M_{0}-M_{E}\right)*e^{-kt}\right]=\left[Ln\left[-k\left(M_{0}-M_{E}\right)\right]-kt\right]=b+mt$$

Therefore $tan\emptyset_{i}=k_{i}=m$ and $e^{b}=\frac{1}{k\left(M_{0}-M_{E}\right)}$.

Evaluating the second Fick’s law solution when n=1 then:

(22) $$M_{R}=e^{-kt}=1+\frac{6}{\pi^{2}}\;e^{\left(-\frac{D_{eff}\pi^{2}t}{a^{2}}\right)}\approx \;\frac{6}{\pi^{2}}\;e^{\left(-\frac{D_{eff}\pi^{2}t}{a^{2}}\right)}\;\;$$

(23) $$Ln\left[M_{R}\right]=Ln\left[e^{-kt}\right]=-kt=Ln\frac{6}{\pi^{2}}-\frac{D_{eff}\pi^{2}t}{a^{2}}=-b-mt$$

(24) $$D_{eff}=\frac{ka^{2}}{\pi^{2}}\;k=\frac{\;\pi^{2}\;D_{eff}}{a^{2}}$$

Employing k value into Equation (9) then:

(25) $$M\left(t\right)=\left(M_{0}-M_{E}\right)e^{-\frac{\;\;\pi^{2}D_{eff}}{a^{2}}t}+M_{E}$$

Then, the moisture rate is calculating as a function of k. In this work, k is named Diffusivity Rate. Therefore:

(26) $$v_{k}=\frac{dM\left(t\right)}{dk}=\frac{dM\left(t\right)}{dt}*\frac{dt}{dk}=\frac{\frac{dM\left(t\right)}{dt}}{\frac{dk}{dt}}=\frac{\left[M_{0}-M_{E}\right]e^{-kt}}{-\frac{1}{t}}=-t\left[M_{0}-M_{E}\right]e^{-\frac{\;\pi^{2}\;D_{eff}}{a^{2}}t}$$

## 4. Results and Discussion

**Figure 1:**
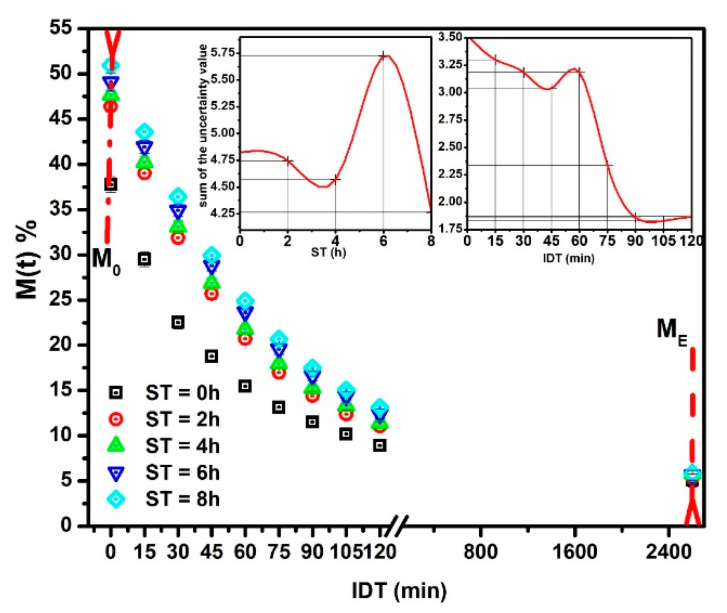
NCG-M(t) experimental points versus IDT for different ST values.

The experimental M(t) versus IDT data (points) for ST = 0, 2, 4, 6 and 8 h are depicted in Figure 1. The reproducibility (*R*^2^) of fitting has a determination value in the 0.994 ≤ *R*^2^ ≤ 0.999 interval. These *R*^2^ values establish the Equation (9) validation to predict the NCG dehydration kinetics as a function of ST. That is, the predicted results will have an uncertainty within the ±0.181–±0.993 interval. All the M(t) versus IDT curves display a similar aspect. The sample with ST = 0, with only cooked alkaline water (black squares), shows a significant difference as compared with others for ST ≠ 0. The initial moisture $M_{0}$ is lower for ST = 0 because, with only cooking time, the water absorbed is less. When ST increases, $M_{0}$ increases significantly [22,23]. M(t) vs. IDT results show similar curves to those reported by [24,25]. The insets in Figure 1 indicate that higher ST and IDT, uncertain values M(t) tend to stabilize in lower values. As a consequence, if the ST-IDT couple of data increases, the measurement reproducibility improves. Nevertheless, in general, the tendency to increase is from high to low IDT values. The importance of this statistical experimental information is that the uncertain values are small; thus, each experimental datum is satisfactory, followed by the mathematical model.

**Figure 2:**
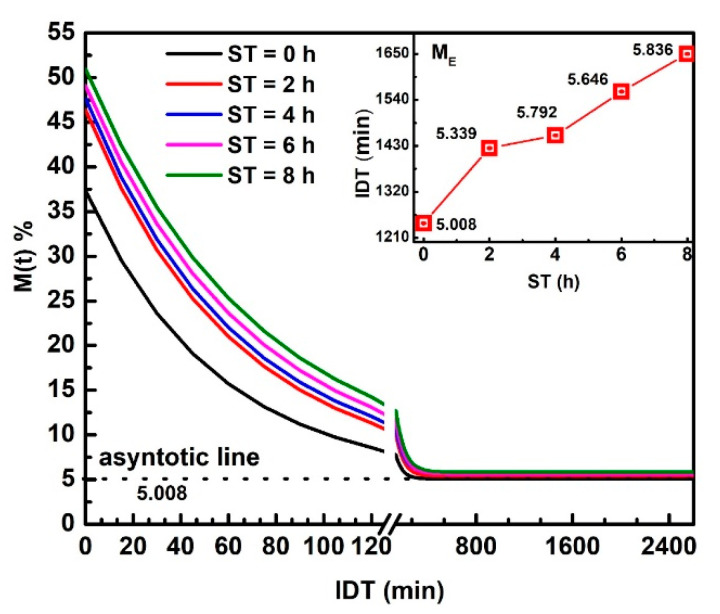
NCG-M(t) fitting as a function of IDT for the ST values studied. The inset exhibits IDT vs. ST, numbers above each point indicate IDT to reach $M_{E}$.

Figure 2 displays M(t) versus IDT curves fitting in the 0 ≤ IDT≤ 2600 min intervals of time. M(t) represents the moisture data within the range from $M_{0}$ up to $M_{E}$.The three $M_{0}$, $M_{E}$_,_ and k, parameters were obtained from the fitting. The inset plots IDT vs. ST; numbers above each point indicate IDT to reach $M_{E}$ for every ST [25]. Notice how IDT to obtain $M_{E}$ increases as ST rises. The IDT versus ST data tendency does not follow smooth increases. The non-smooth, gradual variation of data along a smooth line is not due to errors since they are contained within the size of plotted points. The ST increases have a significant effect on $M_{0}$, which influences the corn masa performance after the NCG milling in a stone mill, affecting the tortillas as a final product. Observe that the moisture tends to be an asymptotic $M_{E}$ value when IDT tends to infinite. $M_{E}$ is the new endogenous moisture limit in all samples, which is similar for all ST and is represented by an asymptotic value in M(t) versus IDT curve.

**Figure 3:**
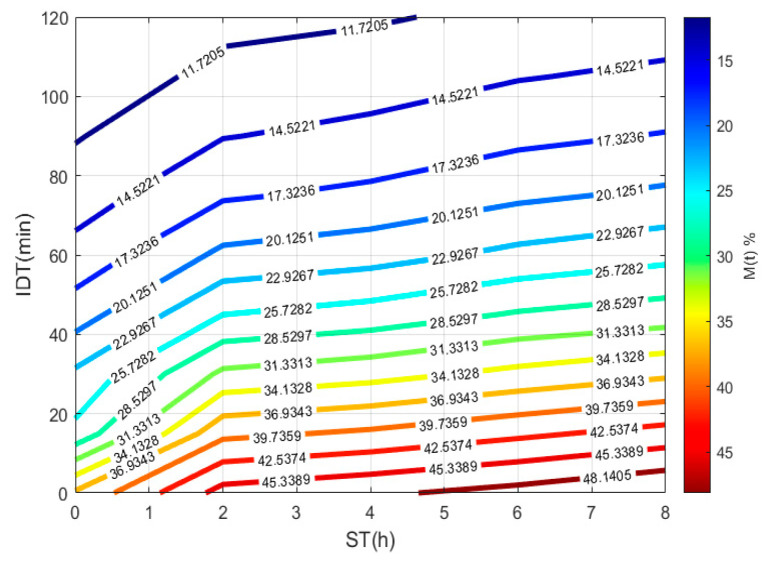
IDT versus ST, where the final product with a specific moisture percentage can be obtained.

To plot Figure 3, a 3D matrix-graph with contour(X,Y,Z,*n*) is used, to obtain moisture percentages isolines, where x = ST, y = IDT, Z = M(t) = moisture constant, and *n* = 14. The exercise practical-technological utility can summarize in (a) needed time prediction for the TNP-NCG process plus dehydration up to moisture needed to prepare the desired product. (b) instantaneous nixtamalized corn flour preparation containing 19–15% moisture to diminish the salmonella growth during storage. (c) instantaneous nixtamalized corn flour moisture percentage standardization for use in labs [25,26,27].

**Figure 4:**
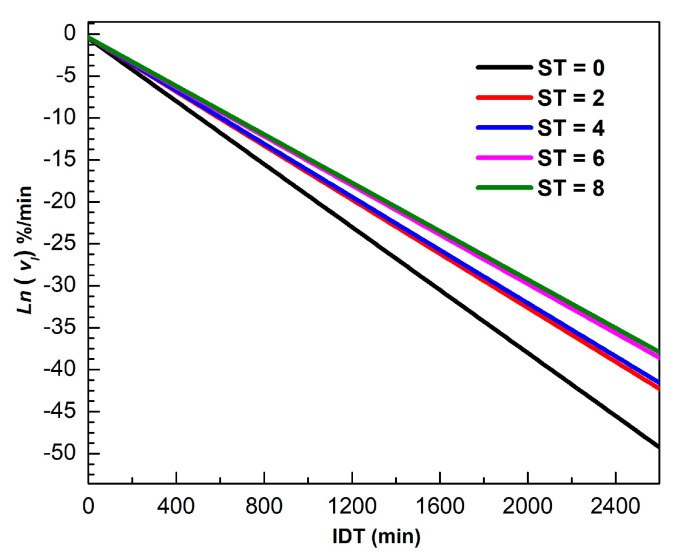
Ln $v_{I}$ versus IDT for different ST.

Figure 4 shows t for different ST of $v_{I}\;$in the 0 ≤ t ≤ 2600 min interval. The logarithm scale in the vertical axis Ln $v_{I}$ facilitates $v_{I}\;$visualization for longer times. The mathematical description of $v_{I}$ is valid for all isothermal dehydration processes, where the slope represents the k dynamical constant. 

**Figure 5:**
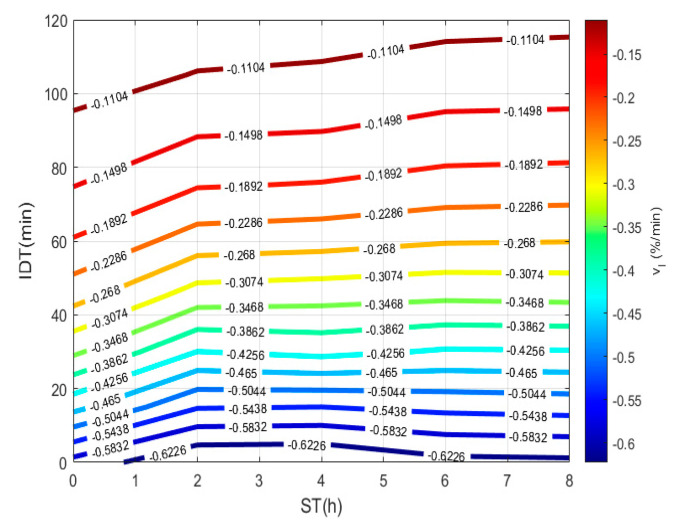
NCG dehydration rate using dehydration time vs. ST.

To plot Figure 5, a 3D matrix-graph with contour(X,Y,Z,*n*) is used to obtain dehydration rate isolines, where x = ST, y = IDT, Z =$\;v_{I}\;$= constant rate and *n* = 14. From data obtained from Equation (11) and listed in Table 1, the rate, in the 0 to 8 h interval, for any ST experimental point can reproduce, and in intermediate points predicted in the 0 to 120 min range. A negative sign physically represents the hydration percentage lost during the NCG dehydration process. The $v_{I}$, which shows a continuous representation during the dehydration process, depends on ST that can be conveniently applied to loss of moisture when being cooked. Thus, corn performance rises to increase the quality of the tortilla.

**Figure 6:**
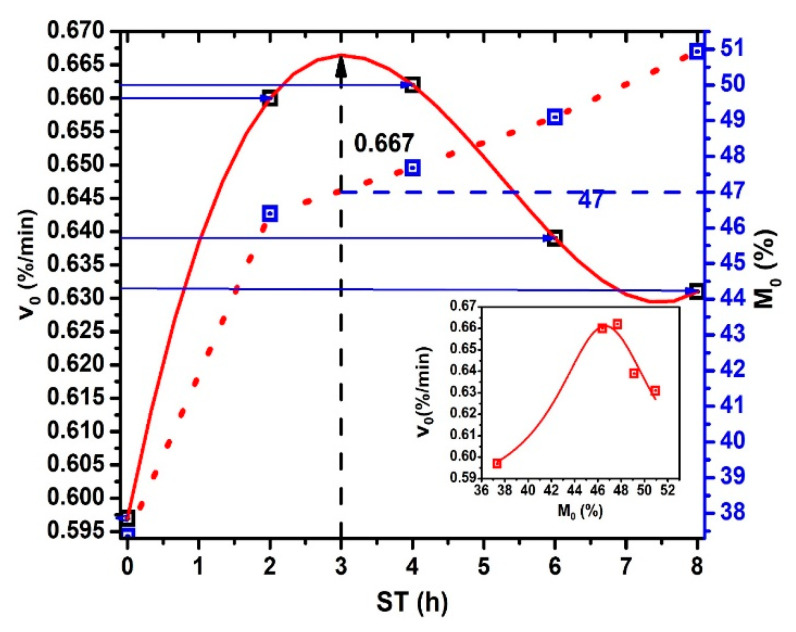
$v_{0}$ and $M_{0}$ vs. *ST*.

The initial dehydration rate versus ST plot shows in Figure 6. Notice that $v_{0}$ obtains when t → 0 and, is calculating for each ST due to k, $M_{0}$, and $M_{E}$ are particular values for each ST. The continuous red line, drawn as an extrapolation, predicts maximum moisture of 47% for ST = 3 h (see the crossing point between the blue dash line and the red dash line projecting by the black dash line until the red extrapolation curve) and decreases like an inverted sigmoid curve in the 3 ≤ ST ≤ 8 interval. This $v_{0}$ values for ST > 3 h can be associated with: (a) increase of both gelatinization and solubilization process of the starches in the NCG periphery. (b) Then, decreasing is the consequence of the gelatinized starch and Ca(OH)^+^- Ca^++^ composite formed during hydrolysis. The Ca(OH)^+^, and Ca^++^ ions effect on the amylose and amylopectin mix in starch is more relevant for molecular crosslinking) [23,26,27,28], (c) there may be increase bonding into calcium ions, amylose, and amylopectin hydroxyl groups in gelatinized starches [29,30], and (d) the decreasing the cracking of NCG during drying. $v_{0}$ also decreases due to a total gelatinization of starch, which shows a radial dependence from the surface to center in the more NCG external layers, as was shown by the lack of different scanning calorimetry profiles generation. [29,30]. When 7 ≤ ST ≤ 8 interval, a relative minimum can be observed. The importance of minimum $v_{0}$ stabilization when ST increases reflect the homogenization and retention of M(t) in the NCG internal structure related to the NCG performance when converted to corn masa. Equal $v_{0}$ values observed in the curve for different moisture percentages and ST values. These behaviors reflect structural properties in the NCG periphery, which modulate $v_{0}$, and indicate the possible presence of pericarp fragments for low ST values. In this way, the total or chemical partial degradation (porous formation) of the superficial pericarp area during TNP is revealed by the physical-chemical analysis of nejayote [30,31]. The $M_{0}$ increasing when ST increases strongly relates to pericarp chemical degradation during the TNP. The inset figure shows the relationship between $v_{0}$ vs. $M_{0}$. Lorentz’s profile is very clear.

**Figure 7:**
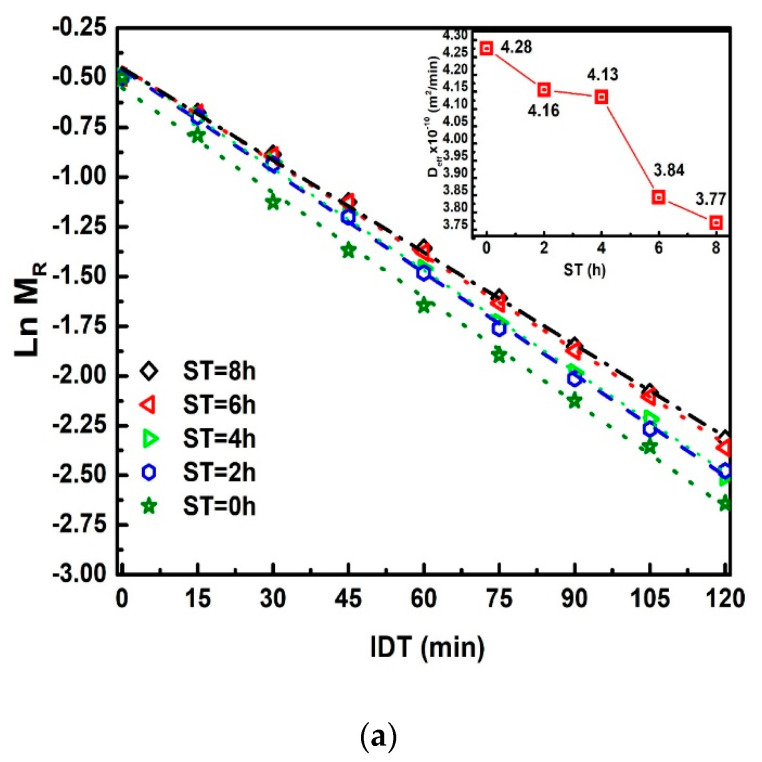

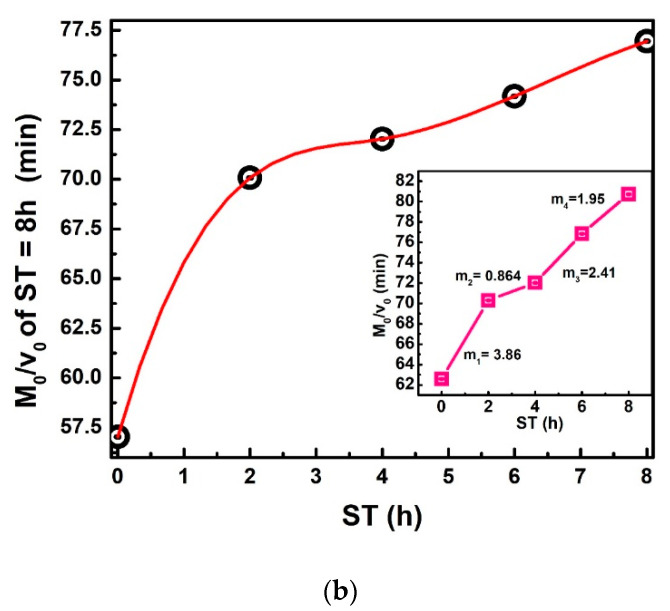
(**a**) $M_{R}$ vs. ST, The inset exhibits 𝐷𝑒𝑓𝑓 vs. ST. (**b**) $M_{0}$**/**$v_{0}$ vs. ST. The red line has been drawn as an approximation.

To plot Figure 7a, Equation (23) is used. The relevance of this equation rests on the comparison of two straight-line equations with a negative slope. The first equals k, and the second is directly proportional to $D_{eff}$, where m=k and is the slope in straight lines and *a* = 0.0049 m; the average radius of the corn grain [30,31]. The practical value of $D_{eff}$ obtains between 2.56 × 10^−6^ to 2.26 × 10^−6^ m^2^/h. The $D_{eff}$ decreases when *ST* increases, as can be observed in the inset of Figure 7a. Moreover, the $D_{eff}$ decreasing can have a direct correlation with the calcium percentage increase when *ST* also increases, which modifies the NCG structural properties on different parts of the grain (pericarp, tip-cap, germ, and endosperm). The ratio $M_{0}$**/**$v_{0}$ versus *ST* is displayed in Figure 7b, $v_{0}$ is the same value as that dividing the dimensionless drying rate [20,32]. This Figure ideally represents the IDT required for dehydration of the total grain percentage moisture $M_{0}$. This red curve does not show a discontinuous slope where the points join through straight lines with a variable slope (*m*_1_, *m*_2_, *m*_3_, *m*_4_), $M_{0}$**/**$v_{0}$ increases when *ST* increases. Furthermore, these increases have the same proportion.

**Figure 8:**
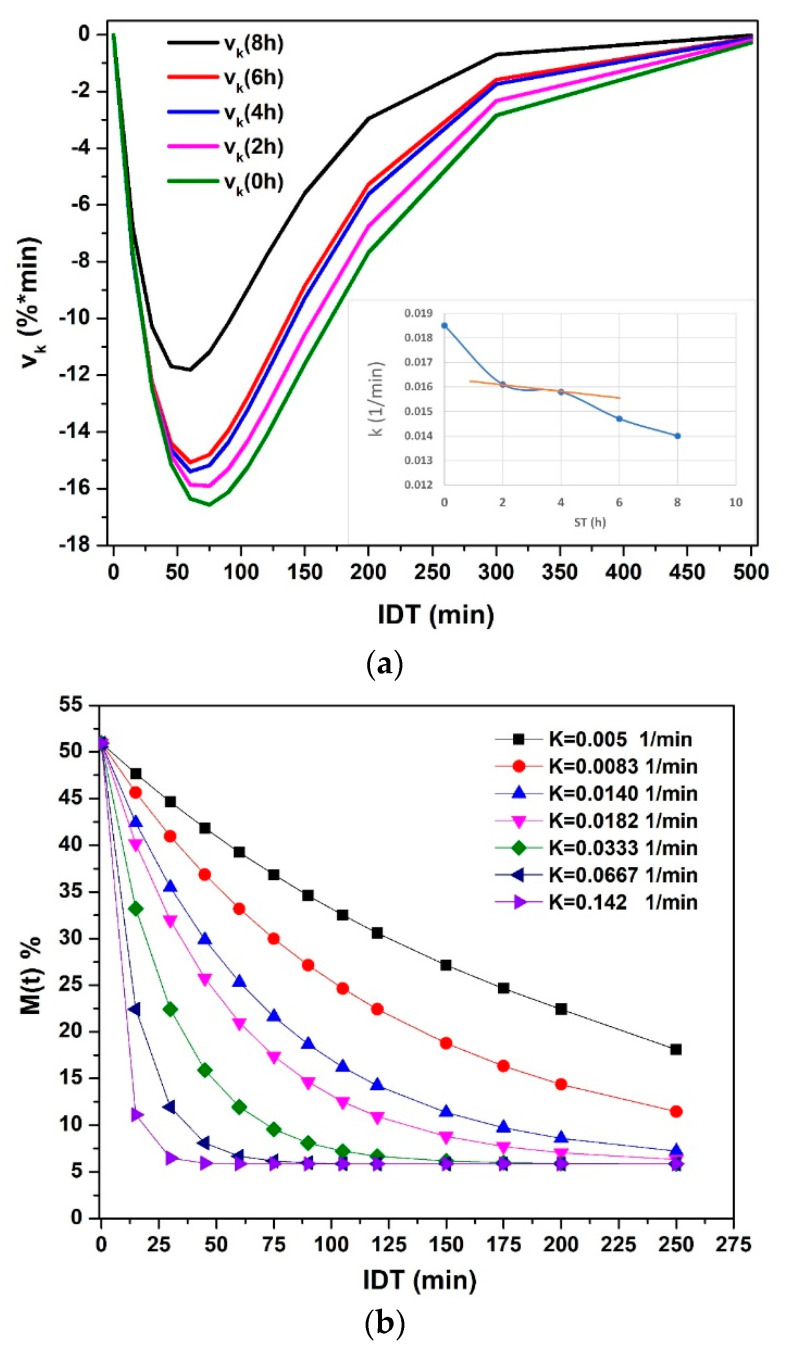
(**a**) The diffusivity rate vs. IDT. (**b**) The moisture percentage vs. IDT with the variation of k for *ST* = 8 h.

Figure 8a plots $v_{k}$ named in this manuscript as the isothermal diffusivity rate; this value represents the variation of k for any t value resulting from the first derivative of the values of k, and that it is directly proportional to the $\;D_{eff}$ and inversely proportional to the square off of the grain surface. Before 100 min, a maximum value of change in the moisture present in the corn grains obtains. When the ST is greater, the maximum loss of moisture is achieved in a long time. A shift to the right indicates that diffusivity slows down due to the bonds of water, and the hydroxyl groups of amylose and amylopectin occur at higher bond energy between them by means of the Van Der Valls forces and a greater number of links [18,26,33,34,35]. After 300 min of drying, the behavior becomes asymptotic with the abscissa axis. The pericarp thickness degradation process is represented by the gap between NCG with/without ST, which is maintained from 0 to 200 min [19,34,36]. Figure 8b. Hypothetical values k are plotted as a simulation of the change over time, knowing that k changes during the ST because the solubility of Ca(OH)_2_ is greater when the temperature of the nejayote decreases. Analyzing Figure 8b, when k increases, the NCG capacity to retain water molecules decreases. From another point of view, when k decreases, the water retention capacity increases. Then the H^+^ and ^-^OH bridges of amylose and amylopectin (from being crystalline to amorphous) with water increase significantly, caused by the physical-chemical process of crosslinking during the gelatinization of the starches on the outermost surface of the NCG [18,19,23,26,27,34,35,36,37,38,39,40,41,42,43,44]. In conclusion, the curvature of the graphed functions indicates that when k increases, the number of free water molecules in the interior structures of the NCG is greater, and it evaporates quicker than the water.

## 5. Conclusions

The most important conclusions of this manuscript are: Firstly, a first-order differential equation describes dehydration kinetics on NCG and, usable in educational texts. Secondly, the mathematical model predicts the necessary moisture percentage and dehydration rate $v_{I}$ of NCG through isolines. Thirdly, when $v_{0}>3$ h of ST decreases although $M_{0}$ increases, thus these values benefit obtaining a better tortilla yield. Fourth, when k and $D_{eff}$ decreases when ST increases. Fifth, the lime solubility into nejayote for different ST has a very strong relationship with the diffusivity rate $v_{k}$ values on NCG. These can have important technological applications such as: (a) milling of NCG in different moisture percentages (dry milling < 15%, 45% < humid milling < 15% and with plenty of water > 45%) according to selected isolines, (b) to avoid the growth of pathogenic organisms’ moisture < 15%, (c) better manufacturing, handling and storage control on various products (snacks, chips, toasts, flours, etc.). Also, it can improve the standardization of the organoleptic and performance properties of the tortilla, the extraction of starches with different alkaline treatments, and carrying out measurements in laboratories, among other known processes. In addition, the manufacturing costs can reduce significantly. It is important to note that the future for the tortilla industry is in the ascendency due to the vast Latin-American emigration to the USA. However, the fifty million Latins still follow their traditional eating habits of which tortillas are an essential part.

## Figures and Tables

**Table 1:** Isothermal Dehydration Rate. Equations for every ST.

ST (h)	$\frac{dM\left(t\right)}{dt}=-k\left[M\left(t\right)-M_{E}\right]$	*R* ^2^
0	$\frac{dM\left(t\right)}{dt}=-0.0185\left[M\left(t\right)-5.088\right]$	0.994
2	$\frac{dM\left(t\right)}{dt}=-0.0161\left[M\left(t\right)-5.399\right]$	0.997
4	$\frac{dM\left(t\right)}{dt}=-0.0158\left[M\left(t\right)-5.792\right]$	0.999
6	$\frac{dM\left(t\right)}{dt}=-0.0147\left[M\left(t\right)-5.646\right]$	0.996
8	$\frac{dM\left(t\right)}{dt}=-0.0140\left[M\left(t\right)-5.836\right]$	0.995

## Data Availability

Not applicable.

## Nomenclature

ST	Steeping Time (hours)
NCG	Nixtamalized Corn Grains
IDT	Isothermal Dehydration Time (minutes)
TNP	Traditional Nixtamalization Process
*a*	meters: average corn grains radius
Ca(OH)_2_	% calcium hydroxide or lime S_X_: uncertainty. Dimensionless
$D_{eff}$	m^2^/min, effective diffusivity coefficient
k	1/min, dynamical proportionality constant (time).
$k_{h}$	1/min, dynamical proportionality constant (time and h=1,2,3,…).
U.S.	mesh open size
M(t)	(%) percentage moisture at t
$M_{0}$	initial moisture (%)
$M_{E}$	(%) equilibrium moisture percentage
$M_{S}$	(%) saturate moisture
$M_{R}$	moisture ratio
$v_{0}$	%/min, Initial Dehydration Rate
$v_{I}$	%/min, Isothermal Dehydration Rate
$v_{k}$	%*min, Diffusivity Rate
t	time (minutes)
isolines	are color lines with the same value but with different simultaneous IDT and ST values.
